# Columbia-Suicide Severity Rating Scale Screen Version: initial screening for suicide risk in a psychiatric emergency department

**DOI:** 10.1017/S0033291721000751

**Published:** 2022-12

**Authors:** Johan Bjureberg, Marie Dahlin, Andreas Carlborg, Hanna Edberg, Axel Haglund, Bo Runeson

**Affiliations:** 1 Department of Clinical Neuroscience, Centre for Psychiatry Research, Karolinska Institutet, & Stockholm Health Care Services, Region Stockholm, Stockholm, Sweden; 2 Department of Psychology, Stanford University, Stanford, California, USA; 3 Department of Clinical Neuroscience, Centre for Psychiatry Research, Karolinska Institutet, & Stockholm Health Care Services, Region Stockholm, Norra Stockholms Psykiatri, Stockholm, Sweden; 4 Department of Clinical Neuroscience, Centre for Psychiatry Research, Karolinska Institutet, & The National Board of Forensic Medicine, Stockholm, Sweden

**Keywords:** Receiver operating characteristic curves, risk assessment, screening, suicide

## Abstract

**Background:**

Suicide screening is routine practice in psychiatric emergency (PE) departments, but evidence for screening instruments is sparse. Improved identification of nascent suicide risk is important for suicide prevention. The aim of the current study was to evaluate the association between the novel Colombia Suicide Severity Rating Scale Screen Version (C-SSRS Screen) and subsequent clinical management and suicide within 1 week, 1 month and 1 year from screening.

**Methods:**

Consecutive patients (*N* = 18 684) attending a PE department in Stockholm, Sweden between 1 May 2016 and 31 December 2017 were assessed with the C-SSRS Screen. All patients (52.1% women; mean age = 39.7, s.d. = 16.9) were followed-up in the National Cause of Death Register. Logistic regression and receiver operating characteristic curves analyses were conducted. Optimal cut-offs and accuracy statistics were calculated.

**Results:**

Both suicidal ideation and behaviour were prevalent at screening. In total, 107 patients died by suicide during follow-up. Both C-SSRS Screen Ideation Severity and Behaviour Scales were associated with death by suicide within 1-week, 1-month and 1-year follow-up. The optimal cut-off for the ideation severity scale was associated with at least four times the odds of dying by suicide within 1 week (adjusted OR 4.7, 95% confidence interval 1.5–14.8). Both scales were also associated with short-term clinical management.

**Conclusions:**

The C-SSRS Screen may be feasible to use in the actual management setting as an initial step before the clinical assessment of suicide risk. Future research may investigate the utility of combining the C-SSRS Screen with a more thorough assessment.

## Background

Suicide accounts for 1.5% of all deaths worldwide (Naghavi & Collaborators, 2019). Suicidal ideation and behaviour are strongly related to psychiatric problems throughout the life cycle (Fazel & Runeson, 2020). People who self-harm have a particularly high rate of eventual suicide, almost 4% die by suicide within the next 5 years (Olfson et al., 2017). Can we prevent suicide by assessing and managing the suicide risk adequately? None of the available assessments to identify patients with suicide risk have a sufficient predictive value (Carter et al., 2018; Fazel & Wolf, 2017; Large et al., 2018; Quinlivan et al., 2016). Shorter actuarial tools or screening instruments for suicide risk may be based on a few items of risk indicators often including previous self-harm, previous psychiatric disorder, or previous psychiatric care contact. Such scales have been studied in emergency departments and aim at improving the decision making if to refer a patient of high risk to psychiatric consultation (Steeg et al., 2012). Other screening instruments mainly based on items of suicidal ideation have also been introduced as an initial screening tool for use in emergency departments (DeVylder et al., 2020; Posner et al., 2011). The initial screening should then be followed by a thorough clinical assessment as suggested by the Zero Suicide Initiative (Brodsky, Spruch-Feiner, & Stanley, 2018; Labouliere et al., 2018). Implementing universal screening within an emergency care setting could twofold increase the number of patients identified within usual care (Boudreaux et al., 2016). However, most studies have only included long follow-up periods (6 months to 5 years), which impedes the clinical utility of the findings, and the relative rarity of death by suicide makes it generally difficult to predict. Thus, based on current evidence, clinical guidelines, such as the NICE guidelines, advice against using risk assessment scales to predict future suicide (Kendall, Taylor, Bhatti, Chan, & Kapur, 2011). Increasing the evidence-base for structured screening of suicide risk has been identified as a research priority (Fazel & Runeson, 2020; Gordon, Avenevoli, & Pearson, 2020).

The Columbia-Suicide Severity Rating Scale (C-SSRS) has been suggested as a routine instrument for use in screening (Posner et al., 2011) and has been evaluated concerning predictive value in both adolescents and adults (Gipson, Agarwala, Opperman, Horwitz, & King, 2015; Lindh et al., 2018; Madan et al., 2016). A short version for initial screening purposes attempting to assess suicidal ideation severity and suicidal behaviours has been introduced, but has to our knowledge not been scientifically evaluated. We aimed at studying the use of the C-SSRS Screen in a psychiatric emergency (PE) department. Is the screening score associated with the risk of suicide within 1-week, 1-month and 1-year of follow-up? Is the C-SSRS Screen associated with the subsequent clinical management of the patient? We implemented the C-SSRS Screen as a part of the initial intake routine and triage of patients at a large PE department. We intended to study the outcome in terms of how suicidal ideation and behaviour were associated with subsequent clinical management, and how the instrument behaved in classifying patients as high or low risk of suicide by calculating accuracy statistics such as sensitivity and specificity of the scale.

## Methods

### Setting and participants

The study cohort included 18 684 consecutive patients attending a psychiatric assessment for any reason at the only PE department in Stockholm, Sweden between 1 May 2016 and 31 December 2017.

### Measurement

Data were extracted from electronic medical records and linked to the National Cause of Death Register held by The National Board of Health and Welfare.

### Predictors

#### Screening of suicidality

The C-SSRS Screen is a structured interview based on the more comprehensive full-length version (Posner et al., 2011). The scale is designed to measure suicidal ideation severity and suicidal behaviours with two subsets of items. The first subset captures past-month severity of suicidal ideation (referred to as the ‘ideation severity scale’). The ideation severity scale is rated on a five-point ordinal scale in which 1 = wish to be dead (‘Have you wished you were dead or wished you could go to sleep and not wake up?’), 2 = non-specific active suicidal thoughts (‘Have you actually had any thoughts of killing yourself?’), 3 = suicidal thoughts with methods (‘Have you been thinking about how you might do this?’), 4 = suicidal intent (‘Have you had these thoughts and had some intention of acting on them?’) and 5 = suicidal intent with plan (‘Have you started to work out or worked out the details of how to kill yourself? Do you intend to carry out this plan?’). The second subset measures past 3-month presence of actual and aborted suicide attempts (referred to as the ‘behaviour scale’) with one item on a nominal scale (‘Have you ever done anything, started to do anything, or prepared to do anything to end your life?’). All assessors were trained and supervised in the C-SSRS Screen and were instructed to conduct the screening interview with all patients as part of the routine intake assessment. The training (about 1.5 h long) was repeated on several occasions in a group setting to include all staff, both day- and nightshift. Supervision was ensured as a specially trained nurse was available full-time, on an as-needed basis, at the PE department. The implementation of the training and the actual C-SSRS Screen was prepared by the management team in collaboration with the research team, including service user representatives. The C-SSRS Screen scores were extracted from the medical record and in case several ratings existed, the last available was used in the analyses.

### Outcomes

#### Death by suicide

Death by suicide was defined as intentional self-harm (ICD-10 codes X60-84) or an event of undetermined intent (Y10-34) as recorded in the national Cause of Death Register from the start of follow-up to 31 December 2018. Self-inflicted deaths of undetermined intent were included to avoid underestimation of suicides (Neeleman & Wessely, 1997; Runeson, Tidemalm, Dahlin, Lichtenstein, & Langstrom, 2010).

#### Short-term clinical management

Short-term psychiatric inpatient care was defined as having a record of being admitted to a psychiatric inpatient care unit in conjunction with the C-SSRS Screening (admitted ⩽1 day). As a measure of short-term outpatient management, we extracted information from the medical record on whether the participants had an outpatient appointment (including counselling) within 7 days from the C-SSRS Screening.

### Statistical analyses

First, ideation severity and behaviour scales were initially analysed separately, entered as independent variables in separate bivariate logistic regression models to yield odds ratios (OR) with 95% confidence intervals (CI) for suicide [analysed separately for suicides occurring ⩽7, ⩽31 and ⩽365 days post the C-SSRS Screening (the three follow-up periods overlapped in terms of the number of patients who died by suicide)], inpatient care and outpatient care in the total sample and separately for men and women. Second, the ideation severity scale was used to conduct receiver operating characteristics (ROC) curves for each follow-up period. The area under the curve (AUC) with 95% CI was assessed as an aggregated measure of performance across all possible classification thresholds for each follow-up period with death by suicide as the outcome. According to established criteria (Šimundić, 2009), AUC between 0.6 and 0.7 and 0.7 and 0.8 was considered to indicate sufficient and good diagnostic accuracy, respectively. We identified cut-offs for the ideation severity scale that maximized the sum of sensitivity and specificity in this sample and accuracy statistics with 95% CI were calculated for all possible cut-offs. This method maximizes the overall correct diagnosis rate and minimizes the overall misdiagnosis and thus equating the expected cost of misclassifying patients not at risk to the expected cost of misclassifying patients who are at risk (see Kaivanto, 2008). These predictors were entered as independent variables in separate bivariate logistic regression models to yield OR (with 95% CI) for suicide. All logistic regression analyses were first analysed without adjustment and then once more, adjusting for sex and age. Logistic regression analyses and accuracy statistics (see Long, Zhang, & Hsu, 2011) were performed on 50 imputed datasets, generated by multiple imputation by chained equations (Azur, Stuart, Frangakis, & Leaf, 2011; Royston, 2011). This method assumes that data are missing at random. As sensitivity analyses, we reran the logistic regression analyses with ideation severity and behaviour scales analysed separately for suicides occurring ⩽7, ⩽31 and ⩽365 days, inpatient care, and outpatient care in total sample using complete data (see online Supplementary Table S1). Estimates and CI changed slightly; however, the overall results were similar across observed and imputed data. Given these results, we present the analyses based on the multiple imputed data. For descriptive purposes, cross-tabulation of the C-SSRS Screen in relation to suicide based on both complete and multiple imputed data for all follow-up periods is presented in the Supplementary material (see online Supplementary Tables S2 and S3). *p* ⩽ 0.05 were considered statistically significant. Finally, the C-SSRS Screen offer general guidelines for risk formulation and suggest a corresponding level of clinical management. Affirmative responses to item 1 or 2 indicate low risk, 3 indicates moderate risk, and 4, 5 or 6 indicates high risk. The number and proportion of patients being admitted to outpatient and inpatient care in relation to risk category are presented. All statistical analyses were performed with Stata, version 15.1 (StataCorp, College Station, TX, USA).

## Results

### Demographic data

Table 1 gives the demographic and clinical data of the total sample (*N* = 18 684). The median number of PE appointments for the total cohort during follow-up was 1 (interquartile range = 1–2; minimum = 1; maximum = 97).

**Table 1.** Demographic and descriptive characteristics of the study cohort

	Total sample (*N* = 18 684)		Suicide ⩽7 days^a^ (*n* = 13)			Suicide ⩽1 month^a^ (*n* = 30)			Suicide ⩽1 year^a^ (*N* = 107)		
	Mean	s.d.	Mean	s.d.	*p*^b^	Mean	s.d.	*p*^b^	Mean	s.d.	*p*^b^
Age (years)	39.7	16.9	50.5	18.4	0.021	47.0	16.9	0.017	44.6	16.5	0.003
	*n*	%	*n*	%	*p*^b^	*n*	%	*p*^b^	*n*	%	*p*^b^
Sex (women)	9739	52.1	7	53.9	0.901	11	36.7	0.090	40	37.4	0.002
Mental disorder^c^											
Anxiety disorders (F30–48)	6756	36.2	3	23.1	0.326	7	23.3	0.143	38	35.5	0.889
Mood disorders (F30–39)	5345	28.6	1	7.7	0.095	5	16.7	0.148	40	37.4	0.044
Substance use disorders (F10–19)	4314	23.1	2	15.4	0.510	8	26.7	0.642	43	40.2	<0.001
ADHD (F90)	1865	10.0	0	0.0	0.230	2	6.7	0.544	16	15.0	0.085
Autism spectrum disorder (F84–89)	1039	5.6	0	0.0	0.381	1	3.3	0.594	10	9.4	0.087
Psychotic disorders (F20–29)	2280	12.2	0	0.0	0.179	4	13.3	0.850	26	24.3	<0.001
Personality disorders (F60)	1270	6.8	0	0.0	0.330	1	3.3	0.451	11	10.3	0.151

ADHD, attention-deficit hyperactivity disorder.

a The three follow-up periods overlapped in terms of the number of patients who died by suicide.

b *p* Value resulting from significance tests comparing to individuals that did not die by suicide during respective follow-up period.

c Mental disorders were defined as any record of ICD-10 principal disorder registered at inpatient or outpatient care, extracted from the medical record, at any time during the follow-up period. Participants could have none or several principal disorders during follow-up.

In total, 21 (0.1%) patients died within 7 days, of whom 13 (0.07%) died by suicide. Among the 13, 11 (84.6%) deaths were classified as resulting from intentional self-injury (ICD-10 codes X60–84) and 2 (15.4%) deaths as undetermined self-injury (Y10–34). The most common suicide method within 7 days was hanging, strangulation and suffocation [ICD-10 code X70; five patients (38.5%)] followed by jumping from a height [ICD-10 code X80; three patients (23.1%)].

Sixty-eight patients (0.4%) died within 1 month from screening of whom 30 (0.2%) died by suicide [*n* = 23 (76.7%) ICD-10 codes X60–84; *n* = 7 (23.3%) Y10–34]. The most common suicide method during this follow-up period was also hanging, strangulation and suffocation [ICD-10 code X70; 10 patients (33.3%)] followed by poisoning [ICD-10 codes X61–64; two patients (6.7%) and Y10–14; five patients (16.7%)].

Finally, 364 (1.9%) patients died within 1 year from screening, of whom 107 (0.6%) died by suicide [*n* = 81 (75.7%) ICD-10 codes X60–84; *n* = 26 (24.3%) Y10–34]. The most common suicide method during this follow-up period was hanging, strangulation and suffocation [ICD-10 code X70; 30 patients (28.0%)] followed by poisoning [ICD-10 codes X61–64; 18 patients (16.8%) and Y10–14; 21 patients (19.6%)]. A complete report of all suicide methods for each follow-up period (by sex and disorder) is available in online Supplementary Table S4.

### Suicidal ideation severity (past month) and behaviours (past 3 months)

In total, 3864 (20.7%) patients were missing ratings of either the C-SSRS Screen ideation severity scale (3462; 18.5%) or the behavioural scale (3687; 19.7%). As presented in online Supplementary Table S5, patients with missing ratings on the C-SSRS Screen were on average 2.4 years older, more likely to be of male sex, having substance use disorder and psychotic disorder, and less likely to have an anxiety disorder, mood disorder, attention-deficit hyperactivity disorder, autism spectrum disorder and personality disorder compared to patients with complete data (these variables were included as auxiliary variables in the multiple imputation). Table 2 shows complete ratings of each item of both scales.

**Table 2.** Frequency for each item of the suicidal ideation severity (past 1 month) and behaviour (past 3 months) scales according to Columbia Suicide Severity Rating Scale Screen Version

	Ideation severity scale	
	*N*	%
Past month		
No suicidal ideation	6545	43.0
Wish to be dead	2333	15.3
Non-specific active suicidal thoughts	2568	16.9
Suicidal thoughts with methods	1668	11.0
Suicidal intent	623	4.1
Suicidal intent with plan	1485	9.8
	Behaviour scale	
	*N*	%
Past 3 months		
No suicidal behaviour	12 670	84.5
Actual and aborted suicide attempts	2327	15.5

### Death by suicide

#### Death by suicide within 1 week

When the follow-up was set to ⩽7 days after the PE appointment, the average number of days between the C-SSRS Screen and suicide was 3.4 ( = 2.1) days (median = 4; minimum = 0; maximum = 7). The ideation severity scale was associated with suicide with an OR of 1.5 (95% CI 1.1–2.1; adjusted OR 1.6, 95% CI 1.2–2.1) for every one-point increase on the scale. The corresponding OR among women was 1.4 (95% CI 0.9–2.1; adjusted OR 1.5, 95% CI 1.0–2.3) and among men 1.7 (95% CI 1.1–2.6; adjusted OR 1.7, 95% CI 1.1–2.6). The behaviour scale predicted suicide with an OR of 6.1 (95% CI 1.9–20.0; adjusted OR 6.9, 95% CI 2.1–22.7). The corresponding OR among women was 6.7 (95% CI 1.2–38.3; adjusted OR 8.6, 95% CI 1.5–50.1) and 5.9 (95% CI 1.1–27.7; adjusted OR 5.8, 95% CI 1.2–28.6) among men.

**Fig. 1.**
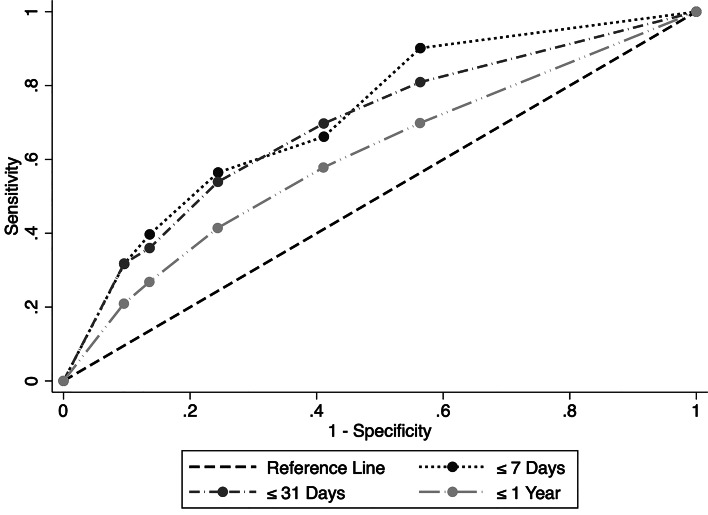
ROC curve for the Columbia Suicide Severity Rating Scale Screen Version: Ideation Severity Scale, predicting suicide during follow-up ⩽7 days [area under the curve (AUC) = 0.72, 95% confidence interval (CI) 0.58–0.86, *p* < 0.001], ⩽31 days (AUC = 0.69, 95% CI 0.59–0.79, *p* < 0.001) and 1 year (AUC = 0.62, 95% CI 0.52–0.72, *p* < 0.001) after C-SSRS Screen rating.

The ROC curve for all follow-up periods is presented in Fig. 1. The AUC for the ideation severity scale (⩽7 days follow-up) was 0.72 (95% CI 0.58–0.86, *p* < 0.001). The optimal cut-off for suicide was ⩾3 (3 = suicidal thoughts with methods), giving a sensitivity of 56.5% (95% CI 53.7–59.2) and a specificity of 75.6% (95% CI 75.5–75.6). This cut-off was associated with an OR of 4.0 (95% CI 1.3–12.6; adjusted OR 4.7, 95% CI 1.5–14.8) for subsequent suicide (Table 3). See online Supplementary Table S6 for accuracy statistics and OR for all cut-offs (for all follow-up periods).

**Table 3.** Accuracy statistics and effect sizes with 95% confidence intervals (CI) for cut-off score ⩾3 of the Columbia Suicide Severity Rating Scale Screen Version: Suicidal Ideation Severity Scale (past 1 month), predicting suicide for ⩽7 days, ⩽31 days and ⩽1 year follow-up

Follow-up/cut-off	Sensitivity (95% CI)	Specificity (95% CI)	Likelihood ratio (+) (95% CI)	Likelihood ratio (−) (95% CI)	PPV (95% CI)	NPV (95% CI)	Crude odds ratio (95% CI)	Adjusted odds ratio (95% CI)^a^
⩽7 days								
⩾3	56.5 (53.7–59.2)	75.6 (75.5–75.6)	2.3 (2.2–2.4)	0.6 (0.5–0.6)	0.2 (0.1–0.2)	100.0 (100.0–100.0)	4.0 (1.3–12.6)	4.7 (1.5–14.8)
⩽31 days								
⩾3	53.9 (52.1–55.7)	75.6 (75.5–75.7)	2.2 (2.1–2.3)	0.6 (0.6–0.6)	0.4 (0.3–0.4)	99.9 (99.9–99.9)	3.6 (1.7–7.7)	4.0 (1.9–8.6)
⩽1 year								
⩾3	41.4 (40.5–42.4)	75.6 (75.6–75.7)	1.7 (1.7–1.7)	0.8 (0.8–0.8)	1.0 (0.9–1.0)	99.6 (99.5–99.6)	2.2 (1.4–3.4)	2.4 (1.5–3.6)

PPV, positive predictive value; NPV, negative predictive value.

a Adjusted for sex and age.

#### Death by suicide within 1 month

For follow-up within ⩽31 days, the number of days between C-SSRS Screen and suicide was on average 11.8 (s.d. = 9.4) days (median = 9; minimum = 0; maximum = 31). The ideation severity scale predicted suicide with an OR of 1.5 (95% CI 1.2–1.8; adjusted OR 1.5, 95% CI 1.2–1.8). The corresponding OR among women was 1.8 (95% CI 1.3–2.6; adjusted OR 2.0, 95% CI 1.4–2.8) and among men 1.3 (95% CI 1.0–1.7; adjusted OR 1.3, 95% CI 1.0–1.7). The behaviour scale predicted suicide with an OR of 4.7 (95% CI 2.1–10.3; adjusted OR 5.1, 95% CI 2.3–11.2). The corresponding OR among women was 9.1 (95% CI 2.3–35.2; adjusted OR 11.4, 95% CI 2.9–45.1) and 3.2 (95% CI 1.2–8.8; adjusted OR 3.3, 95% CI 1.2–9.1) among men.

The AUC for the ideation severity scale was 0.69 (95% CI 0.59–0.79, *p* < 0.001; see Fig. 1) for suicide within a follow-up period of 31 days. The optimal cut-off was ⩾3 points, with a sensitivity of 53.9% (95% CI 52.1–55.7) and a specificity of 75.6% (95% CI 75.5–75.7). This score was associated with an OR of 3.6 (95% CI 1.7–7.7; adjusted OR 4.0, 95% CI 1.9–8.6) for subsequent suicide (see Table 3).

#### Death by suicide within 1 year

Among patients that died within 365 days from screening, the average number of days between C-SSRS Screen and suicide was 125.4 (s.d. = 104.3) days (median = 105; minimum = 0; maximum = 364). The ideation severity scale predicted suicide with an OR of 1.3 (95% CI 1.1–1.4; adjusted OR 1.3, 95% CI 1.1–1.4). The corresponding OR among women was 1.3 (95% CI 1.1–1.6; adjusted OR 1.4, 95% CI 1.2–1.7) and among men 1.2 (95% CI 1.0–1.4; adjusted OR 1.2, 95% CI 1.1–1.4). Analysed separately across different diagnostic groups, point estimates of OR for the ideation severity scale ranged from 1.1 to 1.3 (see online Supplementary Table S7). The behaviour scale predicted suicide with an OR of 2.6 (95% CI 1.6–4.2; adjusted OR 2.8, 95% CI 1.7–4.5). The corresponding OR among women was 3.3 (95% CI 1.6–6.8; adjusted OR 3.7, 95% CI 1.8–7.7) and 2.3 (95% CI 1.2–4.1; adjusted OR 2.3, 95% CI 1.3–4.3) among men. Point estimates of OR for the behaviour scale ranged from 0.9 (for autism spectrum disorder) to 3.7 (for psychotic disorders) across different diagnostic groups (see online Supplementary Table S7).

The AUC for the ideation severity scale for suicide within a follow-up period of 365 days was 0.62 (95% CI 0.52–0.72, *p* < 0.001; see Fig. 1). The optimal cut-off was ⩾3 points, with a sensitivity of 41.4% (95% CI 40.5–42.4) and a specificity of 75.6% (95% CI 75.6–75.7). This cut-off was associated with an OR of 2.2 (95% CI 1.4–3.4; adjusted OR 2.3, 95% CI 1.5–3.6) for subsequent suicide (see Table 3).

### Short-term clinical management

Half of the sample (*n* = 9362; 50.1%) were admitted to psychiatric inpatient care at least once during follow-up, of whom 6848 (73.1%) admissions were committed in direct conjunction with the C-SSRS Screen rating. The ideation severity scale was associated with immediate admission with an OR of 1.2 (95% CI 1.2–1.3; adjusted OR 1.3, 95% CI 1.2–1.3). The corresponding OR among women was 1.2 (95% CI 1.2–1.3; adjusted OR 1.3, 95% CI 1.3–1.3) and among men 1.2 (95% CI 1.2–1.2; adjusted OR 1.2, 95% CI 1.2–1.3). Similarly, the behaviour scale was associated with immediate inpatient care with an OR of 2.5 (95% CI 2.3–2.7; adjusted OR 2.7, 95% CI 2.5–3.0). The corresponding OR among women was 2.5 (95% CI 2.2–2.8; adjusted OR 2.8, 95% CI 2.5–3.2) and 2.5 among men (95% CI 2.2–2.8; adjusted ORs 2.6, 95% CI 2.3–3.0).

Almost half of the entire sample (*n* = 9150; 49.0%) visited a psychiatric outpatient clinic within 7 days of the C-SSRS Screen assessment. The ideation severity scale predicted outpatient visits (⩽7 days) with an OR of 1.2 (95% CI 1.2–1.3; adjusted OR 1.3, 95% CI 1.2–1.3). The corresponding OR among women was 1.3 (95% CI 1.2–1.3; adjusted OR 1.3, 95% CI 1.3–1.3) and among men 1.2 (95% CI 1.2–1.3; adjusted OR 1.2, 95% CI 1.2–1.3). Similarly, C-SSRS Screen behaviour scale predicted outpatient visits (⩽7 days) with an OR of 2.5 (95% CI 2.3–2.8; adjusted OR 2.7; 95% CI 2.4–3.0). The corresponding OR among women was 2.6 (95% CI 2.3–3.0; adjusted OR 2.9, 95% CI 2.5–3.2) and 2.4 among men (95% CI 2.1–2.8; adjusted OR 2.5, 95% CI 2.2–2.9).

Among the 8433 patients who were categorized as low risk (according to the C-SSRS Screen general guidelines), 3396 (40.3%) were admitted to psychiatric outpatient care within 7 days and 2384 (28.3%) were immediately admitted to inpatient care. In total, 2225 patients were categorized as moderate risk, of which 1011 (45.4%) were admitted to psychiatric outpatient care within 7 days and 659 (29.6%) were admitted to inpatient care. Finally, among the 3817 who were categorized as high risk, 2467 (64.6%) were admitted to psychiatric outpatient care within 7 days and 1943 (50.9%) were admitted to inpatient care.

### C-SSRS ideation severity, admission to inpatient care and death by suicide

Out of the 3776 patients who scored ⩾3 of the data-derived cut-off on the ideation severity scale, 1928 (51.1%) were admitted to inpatient care in conjunction with the C-SSRS Screening. Among these patients, three, nine and 24 died by suicide within 1 week, 1 month and 1 year, respectively. The number of deaths by suicide for patients who scored ⩾3 on the ideation scale but were not admitted to psychiatric inpatient care was four, six and 13. These figures corresponded to a crude OR of 0.7 (95% CI 0.2–3.2), 1.4 (95% CI 0.5–4.1) and 1.8 (95% CI 0.9–3.5).

## Discussion

This prospective cohort study included 18 684 consecutive patients attending a PE department during a period of 20 months. The C-SSRS Screen ideation severity scale had some predictive value in correctly classifying suicides, particularly for immediate suicides (within 1 week and 1 month). Patients scoring over the data-derived cut-off (⩾3; suicidal thoughts with methods) had almost four times the odds of dying by suicide within 1 week and 1 month, respectively, and doubled the odds of dying within a year. In actual numbers, seven (53.8%), 16 (53.3%) and 44 (41.1%) patients who died by suicide had been rated at or above cut-off at respective follow-up period. Further, endorsing recent actual or aborted suicide attempts was associated with four times the odds of dying by suicide within 1 week and 1 month and doubled odds of dying within a year. The C-SSRS Screen was associated with both immediate inpatient care and follow-up psychiatric outpatient management and the majority of high-risk patients (according to the C-SSRS Screen general guidelines) were appropriately immediately admitted to inpatient care and were followed-up within outpatient care within 7 days. However, our results may indicate – albeit not statistically significant – that although inpatient care might provide immediate protection from death by suicide within a very brief time period (i.e. 1 week), inpatient care was not associated with decreased risk of suicide within 1 month or 1 year, on contrary absolute risks and the size of OR may indicate that inpatient care could be an indicator of increased risk of suicide within 1 month and 1 year.

This is the first study to provide evidence for the abbreviated C-SSRS Screen version, and also to investigate how the C-SSRS ideation severity scale is associated with subsequent death by suicide (not only attempted suicide; Gipson et al., 2015; Katz, Barry, Cooper, Kasprow, & Hoff, 2019; Lind et al., 2018; Madan et al., 2016; Posner et al., 2011). Our findings that the behaviour scale was associated with suicide align well with robust evidence that a history of suicide attempt is associated with subsequent suicide (Olfson et al., 2017; Runeson, Haglund, Lichtenstein, & Tidemalm, 2015). In line with current thinking (Mou, Kleiman, & Nock, 2020), our findings indicate that suicide risk assessment may benefit from subtyping suicidal thought and behaviour, instead of studying these phenomena as a homogeneous construct. Indeed, suicide risk is a complex phenomenon involving a number of intra- and interindividual factors that interact with each other (Fazel & Runeson, 2020). Thus, although the C-SSRS Screen may capture different phenotypes of suicidal thinking and behaviour, any single screening measure will have limited predictive validity. Indeed, although some test statistics detected in the present study indicate the clinical utility of the C-SSRS Screen, the positive predictive value (PPV; the proportion of patients identified as high-risk who die by suicide) for death by suicide was generally low for the different cut-off scores (⩽1.2%; see online Supplementary Table S6). This is within the lower range usually found in the evaluation of suicide prediction models (Barak-Corren et al., 2017; Belsher et al., 2019; Runeson et al., 2017; Simon et al., 2018). However, when comparing PPV between studies, one must consider the prevalence of the outcome for the particular setting and follow-up time, since the rarity of suicide places a ceiling on the PPV (Belsher et al., 2019). For example, given the low total number of suicides within 1 week in our sample (*n* = 13; 0.07%), our test would only yield a PPV of 1.3%, even with hypothetical optimal test accuracy such as 95% sensitivity and 95% specificity. Still, the incidence in the high-risk group found in the present sample is too low to motivate highly interfering interventions such as admission to inpatient care in all cases. Such recommendation would pose a serious risk for patient integrity and an increased workload for treatment facilities, in cases of false-positive risks of suicide. The negative predictive value (NPV; the proportion of patients identified as low risk who do not die by suicide) was high in the present study. This is expected according to the suicide prediction literature (Belsher et al., 2019; Runeson et al., 2017; Whiting & Fazel, 2019). However, this metric must also be interpreted with caution since NPV may be artificially high with very rare outcomes. Nevertheless, our findings indicate that the C-SSRS Screen can potentially be useful to initiate communication about suicide in a structured manner but any attempt to stratify risk as low or high must also incorporate information about known risk factors (Fazel & Runeson, 2020; Labouliere et al., 2018) and only be used to make decisions where a false-positive rate is acceptable, such as determining who to assess more fully or allocate to a non-invasive treatment option. Such treatment options include developing safety plans (Stanley et al., 2018), phone follow-up (Exbrayat et al., 2017), digital interventions specifically developed to address suicidality (Büscher, Torok, Terhorst, & Sander, 2020; Torok et al., 2020), brief outpatient programmes (e.g. Attempted Suicide Short Intervention Program; Gysin-Maillart, Schwab, Soravia, Megert, & Michel, 2016), intensive outpatient treatment programmes such as cognitive behavioural therapy, including dialectical behavioural therapy, and ensuring timely enrolment in outpatient services should be considered for risk individuals (Zalsman et al., 2016). In line with previous reasoning (Large, Ryan, Carter, & Kapur, 2017), regardless of screening outcome; clinicians should never dismiss any patient as low risk based on C-SSRS Screen if they themselves vocal concerns of suicide. Further, although the C-SSRS Screen (assessing past 1 month ideation severity and past 3 months suicidal behaviour) may increase the chance of identifying individuals who are at immediate risk of suicide within a few days after PE contact, the association of screening score and suicide was lower for longer follow-up periods. This underscores the clinical relevance, in line with previous recommendations, of continuing evaluating methods that assess suicide risk within brief time-periods (Bolton, Gunnel, & Turecki, 2015). Future research may consider comparing short-term screening (e.g. past 1 month suicidal ideation and past 3 months suicidal behaviour) with lifetime screening expecting that short-term screening is better able to predict imminent rather than long-term risk, which would have pivotal relevance for the inclusion of items in abbreviated suicide risk assessments.

Further, our results indicate that although inpatient care might provide protection from death by suicide within 1 week, immediate inpatient care following a score of ⩾3 on the ideation severity scale may be an indication of increased risk of death by suicide within 1 month and 1 year. Although these findings were based on few events and must be interpreted with caution, they are in line with recent studies showing that inpatient care is associated with death by suicide within 1 year (Walter et al., 2017, 2019), which may be the result of confounding by indication and warrant future study. Moreover, there was a tendency that both the ideation severity and behaviour scales were generally stronger associated with death by suicide for women than for men (although not formally tested), a finding that may have implications for C-SSRS Screen utility and further development. Interestingly, few patients endorsed suicidal intent without a plan, suggesting that most psychiatric ED patients with suicidal ideation also have a plan. Another notable finding was that the association between C-SSRS Screen ideation severity and behaviour scale was stronger among patients with a psychotic disorder. This is encouraging considering that it is particularly challenging to predict suicide in this patient group (Lopez-Morinigo et al., 2016). Nevertheless, the C-SSRS Screen seemed to perform in a similar way irrespective of psychiatric disorder.

### Strengths and limitations

There are several strengths to our study. To our knowledge, this is one of the largest natural unselected population-based studies on the association between any version of the C-SSRS and death by suicide. The study was conducted independently of the constructors of the C-SSRS, in another country, with few missing data, and the possibility to study sex differences while controlling for age. Our results provide evidence of significant prospective associations between both suicidal ideation severity and suicidal behaviour with both death by suicide and clinical management by using stringent criteria for exposure and outcome, among patients attending a PE department.

Important limitations include that our study design did not take individual differences in time to follow-up in consideration and did not allow for examination of repeated measure. In addition, we cannot determinate the direct causal relationship between C-SSRS Screen, clinical management and suicide rates. Finally, the study was conducted on PE department patients. The clinicians conducting the screening had clinical training in mental health in addition to specific training in how to conduct the C-SSRS Screen. Moreover, the feasibility and acceptability of asking questions about suicidality may be higher in a specialized psychiatric setting compared to more general medical settings. Thus, it is not clear how the findings would generalize to other services such as medical emergency departments.

## Conclusions

Suicidal ideation of increasing severity and suicide attempts, as measured by the C-SSRS Screen, was associated with death by suicide but also increased clinical management. The presence of suicidal thoughts with a method over the past month and suicidal behaviour over the past 3 months was associated with similar odds (four times) of dying by suicide. C-SSRS Screen may have utility in a clinical management setting to initiate communication about short-term suicide risk in a structured manner among both men and women, and may be useful to identify suicide in the immediate time interval of a few weeks. Implementation of a screening in an emergency department may be justified if the purpose is to alert the psychiatric clinical assessment of making a thorough suicide risk evaluation. Future studies should investigate the utility of combining the C-SSRS Screen with a more thorough assessment.

## Supplementary material

For supplementary material accompanying this paper visit https://doi.org/10.1017/S0033291721000751. click here to view supplementary material
