# Supplementary material for: Columbia-Suicide Severity Rating Scale Screen Version: initial screening for suicide risk in a psychiatric emergency department
Source: Psychol Med. 2021 Mar 26;52(16):3904–12. doi: 10.1017/S0033291721000751 (PMC9811343; doi:10.1017/S0033291721000751)
Supplement: Supplementary file 1 [file S0033291721000751sup001.docx]

**SUPPLEMENTAL MATERIALS**

**Supplement to: Columbia-Suicide Severity Rating Scale Screen Version: Initial Screening For Suicide Risk In A Psychiatric Emergency Department**

Johan Bjureberg, PhD^1,2^, Marie Dahlin, MD, PhD^3^, Andreas Carlborg, MD, PhD, MBA^3^, Hanna Edberg, MD^3^, Axel Haglund, MD, PHD^4^, Bo Runeson, MD, PhD^1^

^1^Centre for Psychiatry Research, Department of Clinical Neuroscience, Karolinska Institutet, & Stockholm Health Care Services, Region Stockholm, Stockholm, Sweden

^2^Department of Psychology, Stanford University, Stanford, California, USA

^3^Centre for Psychiatry Research, Department of Clinical Neuroscience,
Karolinska Institutet, & Stockholm Health Care Services, Region Stockholm,
Norra Stockholms psykiatri, Stockholm, Sweden

^4^Centre for Psychiatry Research, Department of Clinical Neuroscience,
Karolinska Institutet, & The National Board of Forensic Medicine, Stockholm, Sweden

**Correspondence:** Johan Bjureberg, Department of Clinical Neuroscience, Karolinska Institutet,
Norra Stationsgatan 69, floor 7, SE-11364 Stockholm, Sweden. Email: Johan.Bjureberg@ki.se

Running Head: C-SSRS Screen in a psychiatric emergency department

**STable 1. Associations between Columbia Suicide Severity Rating Scale Screen Version: Suicidal Ideation Severity Scale (past 1 month) and Suicide Behavior Scale (past 3 months) and suicide within ≤7 days, ≤31 days, and ≤1 year follow-up and short-term clinical management. Analyses based on complete data. Associations expressed as odds ratios with 95% confidence intervals (CI).**

|  | **Suicidal Ideation Scale** (N=15,222) | | **Behavior Scale** (N=14,997) | |
| --- | --- | --- | --- | --- |
|  | Crude odds ratio  (95% CI) | Adjusted odds ratio (95% CI)^a^ | Crude odds ratio  (95% CI) | Adjusted odds ratio (95% CI)^a^ |
| **Suicide** |  |  |  |  |
| ≤7 days | 1.5 (1.1-2.1) | 1.6 (1.2-2.2) | 6.5 (2.0-21.5) | 7.1 (2.2-23.5) |
| ≤31 days | 1.5 (1.2-1.8) | 1.5 (1.2-1.9) | 5.0 (2.3-11.1) | 5.5 (2.5-12.1) |
| ≤1 year | 1.3 (1.1-1.4) | 1.3 (1.2-1.5) | 2.7 (1.7-4.3) | 2.9 (1.8-4.6) |
| **Short-term Clinical Management** |  |  |  |  |
| Inpatient | 1.3 (1.3-1.3) | 1.3 (1.3-1.4) | 2.9 (2.6-3.2) | 3.2 (2.9-3.5) |
| Outpatient | 1.3 (1.3-1.3) | 1.3 (1.3-1.3) | 2.9 (2.6-3.1) | 3.1 (2.8-3.4) |

^a^ Adjusted for sex and age.

**STable 2. Cross tabulation of observed suicides in relation to the Columbia Suicide Severity Rating Scale Screen Version: Suicidal Ideation Severity Scale (past 1 month) score (0-5) for suicides within ≤7 days, ≤31 days, and ≤1 year follow-up (based on complete data).**

|  | **0** | **1** | **2** | **3** | **4** | **5** |
| --- | --- | --- | --- | --- | --- | --- |
| **≤7 days** |  |  |  |  |  |  |
| No Suicide | 6,544 | 2,330 | 2,567 | 1,666 | 622 | 1,481 |
| Suicide | 1 | 3 | 1 | 2 | 1 | 4 |
| **≤31 days** |  |  |  |  |  |  |
| No Suicide | 6,540 | 2,330 | 2,564 | 1,663 | 622 | 1,476 |
| Suicide | 5 | 3 | 4 | 5 | 1 | 9 |
| **≤1 year** |  |  |  |  |  |  |
| No Suicide | 6,519 | 2,323 | 2,554 | 1,655 | 618 | 1,466 |
| Suicide | 26 | 10 | 14 | 13 | 5 | 19 |

**STable 3. Average cross tabulation of suicides in relation to the Columbia Suicide Severity Rating Scale Screen Version: Suicidal Ideation Severity Scale (past 1 month) score (0-5) for suicides ≤7 days, ≤31 days, and ≤1 year follow-up, across multiple imputed data sets.**

|  | **0** | **1** | **2** | **3** | **4** | **5** |
| --- | --- | --- | --- | --- | --- | --- |
| **≤7 days** |  |  |  |  |  |  |
| No Suicide | 8141.88 | 2846.34 | 3120.22 | 2018.68 | 751.96 | 1791.92 |
| Suicide | 1.28 | 3.12 | 1.26 | 2.18 | 1.04 | 4.12 |
| **≤31 days** |  |  |  |  |  |  |
| No Suicide | 8137.44 | 2846.1 | 3116.74 | 2015.48 | 751.74 | 1786.5 |
| Suicide | 5.72 | 3.3 | 4.74 | 5.38 | 1.2 | 9.54 |
| **≤1 year** |  |  |  |  |  |  |
| No Suicide | 8110.9 | 2836.58 | 3103.94 | 2005.22 | 746.72 | 1773.64 |
| Suicide | 32.26 | 12.88 | 17.54 | 15.64 | 6.28 | 22.4 |

**STable 4. Frequency of suicide methods (International Classification of Diseases, Tenth Revision codes) within 1 year from screening with Columbia Suicide Severity Rating Scale Screen Version by sex and disorder.**

|  | **Total** | **Women**  (%) | **F30-48**  Anxiety (%) | **F30-39**  Mood (%) | **F10-19**  Substance (%) | **F90**  ADHD (%) | **F84-89**  ASD (%) | **F20-29**  Psychotic (%) | **F60**  Personality (%) |
| --- | --- | --- | --- | --- | --- | --- | --- | --- | --- |
| **X70** Hanging, strangulation | 30 | 9 (30.0) | 10 (33.3) | 9 (30.0) | 6 (20.0) | 2 (6.7) | 1 (3.3) | 4 (13.3) | 2 (6.7) |
| **Y10–14** Poisoning | 21 | 10 (47.6) | 8 (38.1) | 6 (28.6) | 15 (71.4) | 3 (14.3) | 1 (4.8) | 2 (9.5) | 3 (14.3) |
| **X61–64** Self-poisoning | 18 | 13 (72.2) | 8 (44.4) | 11 (61.1) | 8 (44.4) | 5 (27.8) | 4 (22.2) | 6 (33.3) | 3 (16.7) |
| **X81** Jumping, moving object | 15 | 2 (13.3) | 5 (33.3) | 7 (46.7) | 8 (53.3) | 3 (20.0) | 2 (13.3) | 6 (40.0) | 3 (20.0) |
| **X80** Jumping from a height | 6 | 2 (33.3) | 1 (16.7) | 1 (16.7) | 0 (0.0) | 0 (0.0) | 0 (0.0) | 1 (16.7) | 0 (0.0) |
| **X78** Sharp object | 5 | 1 (20.0) | 2 (40.0) | 2 (40.0) | 4 (80.0) | 0 (0.0) | 0 (0.0) | 1 (20.0) | 0 (0.0) |
| **X71** Drowning | 3 | 2 (66.7) | 2 (66.8) | 0 (0.0) | 1 (33.3) | 0 (0.0) | 0 (0.0) | 2 (66.7) | 0 (0.0) |
| **X72-73** Firearm | 2 | 0 (0.0) | 1 (50.0) | 2 (100.0) | 1 (50.0) | 2 (100.0) | 1 (50.0) | 1 (50.0) | 0 (0.0) |
| **X76** Smoke/fire | 2 | 0 (0.0) | 1 (50.0) | 1 (50.0) | 0 (0.0) | 1 (50.0) | 0 (0.0) | 2 (100.0) | 0 (0.0) |
| **Y21** Drowning | 2 | 0 (0.0) | 0 (0.0) | 0 (0.0) | 0 (0.0) | 0 (0.0) | 0 (0.0) | 0 (0.0) | 0 (0.0) |
| **Y32-33** Crash | 2 | 1 (50.0) | 0 (0.0) | 1 (50.0) | 0 (0.0) | 0 (0.0) | 0 (0.0) | 0 (0.0) | 0 (0.0) |
| **Y20** Hanging, strangulation | 1 | 0 (0.0) | 0 (0.0) | 0 (0.0) | 0 (0.0) | 0 (0.0) | 1 (100.0) | 1 (100.0) | 0 (0.0) |

ADHD=Attention-deficit hyperactivity disorder; ASD=Autism Spectrum Disorder

**STable 5. Demographic and descriptive characteristics of participants with complete data respective missing data on the C-SSRS Screen.**

|  | **Complete data  (N=14,820)** | | **Missing on C-SSRS  Screen (N=3,864)** | | |
| --- | --- | --- | --- | --- | --- |
|  | Mean | SD | Mean | SD | *p****^a^*** |
| Age (years) | 39.2 | 16.5 | 41.6 | 18.2 | <.001 |
|  |  |  |  |  |  |
|  | n | % | n | % | *p^a^* |
| Sex (Women) | 7,820 | 52.8 | 1,919 | 49.7 | .001 |
| *Mental Disorder^b^* |  |  |  |  |  |
| Anxiety disorders (F30-48) | 5,750 | 38.8 | 1,006 | 26.0 | <.001 |
| Mood disorders (F30-39) | 4,551 | 30.7 | 794 | 20.6 | <.001 |
| Substance use disorders (F10-19) | 3,370 | 22.7 | 944 | 24.4 | .026 |
| ADHD (F90) | 1,561 | 10.5 | 304 | 7.8 | <.001 |
| Autism spectrum disorder (F84-89) | 879 | 5.9 | 160 | 4.1 | <.001 |
| Psychotic disorders (F20-29) | 1,699 | 11.5 | 581 | 15.0 | <.001 |
| Personality disorders (F60) | 1,088 | 7.3 | 182 | 4.7 | <.001 |

ADHD=Attention-deficit hyperactivity disorder.
^a^ *P* Value resulting from significance tests comparing patients with missing data on the C-SSRS Screen to patients with complete data.
^b^ Mental disorders were defined as any record of ICD-10 principal disorder registered at inpatient or outpatient care, extracted from the medical record, at any time during the follow-up period. Participants could have none or several principal disorders during follow-up.

**STable 6. Accuracy statistics and effect sizes with 95% confidence intervals (CI) for different cut-off values of the Columbia Suicide Severity Rating Scale Screen Version: Suicidal Ideation Severity Scale (past 1 month), predicting suicide for ≤7 days, ≤31 days, and ≤1 year follow-up.**

| **Follow-up / Cut-off** | **Sensitivity (95% CI)** | **Specificity**  **(95% CI)** | **Likelihood ratio (+)**  **(95% CI)** | **Likelihood ratio (-)**  **(95% CI)** | **PPV**  **(95% CI)** | **NPV**  **(95% CI)** | **Crude  odds ratio**  **(95% CI)** | **Adjusted odds ratio**  **(95% CI)**^a^ |
| --- | --- | --- | --- | --- | --- | --- | --- | --- |
| **≤7 days** |  |  |  |  |  |  |  |  |
| ≥1 | 90.2  (88.4–91.7) | 43.6  (43.5–43.7) | 1.6  (1.6–1.6) | 0.2  (0.2–0.3) | 0.1  (0.1–0.1) | 100  (100.0–100.0) | 7.5  (1.0–56.9) | 8.8  (1.2–67.9) |
| ≥2 | 66.2  (63.5–68.7) | 58.9  (58.8–58.9) | 1.6  (1.6–1.7) | 0.6  (0.5–0.6) | 0.1  (0.1–0.1) | 100.0  (100.0-100.0) | 2.8  (0.8–9.3) | 3.3  (1.0–11.3) |
| ≥3 | 56.5  (53.7–59.2) | 75.6  (75.5–75.6) | 2.3  (2.2–2.4) | 0.6  (0.5–0.6) | 0.2  (0.1–0.2) | 100.0  (100.0-100.0) | 4.0  (1.3–12.6) | 4.7  (1.5–14.8) |
| ≥4 | 39.7  (37.0–42.4) | 86.4  (86.3–86.4) | 2.9  (2.7–3.1) | 0.7  (0.7–0.7) | 0.2  (0.2–0.2) | 100.0  (99.9–100.0) | 4.2  (1.3–13.0) | 4.7  (1.5–14.8) |
| ≥5 | 31.7  (29.2–34.3) | 90.4  (90.4–90.4) | 3.3  (3.1­–3.6) | 0.8  (0.7–0.8) | 0.2  (0.2–0.3) | 99.9  (99.9–100.0) | 4.4  (1.3–14.4) | 4.8  (1.5–16.1) |
| **≤31 days** |  |  |  |  |  |  |  |  |
| ≥1 | 80.9  (79.5–82.3) | 43.6  (43.6–43.7) | 1.4  (1.4–1.5) | 0.4  (0.4–0.5) | 0.2  (0.2–0.2) | 99.9  (99.9–99.9) | 3.3  (1.3–8.7) | 3.8  (1.4–9.4) |
| ≥2 | 69.7  (68.1–71.4) | 58.9  (58.8–59.0) | 1.7  (1.7–1.7) | 0.5  (0.5–0.5) | 0.3  (0.3–0.3) | 99.9  (99.9–99.9) | 3.3  (1.5–7.5) | 3.7  (1.6–8.6) |
| ≥3 | 53.9  (52.1–55.7) | 75.6  (75.5–75.7) | 2.2  (2.1–2.3) | 0.6  (0.6–0.6) | 0.4  (0.3–0.4) | 99.9  (99.9–99.9) | 3.6  (1.7–7.7) | 4.0  (1.9–8.6) |
| ≥4 | 36.0  (34.3–37.7) | 86.4  (86.3–86.4) | 2.7  (2.5–2.8) | 0.7  (0.7–0.8) | 0.4  (0.4–0.4) | 99.9  (99.9–99.9) | 3.6  (1.6–7.8) | 3.9  (1.8–8.4) |
| ≥5 | 31.8  (30.1–33.5) | 90.4  (90.4–90.5) | 3.3  (3.2–3.5) | 0.8  (0.7–0.8) | 0.5  (0.5–0.6) | 99.9  (99.9–99.9) | 4.4  (2.0–9.7) | 4.7  (2.1–10.3) |
| **≤1 year** |  |  |  |  |  |  |  |  |
| ≥1 | 69.9  (69.0–70.7) | 43.7  (43.6–43.7) | 1.2  (1.2–1.3) | 0.7  (0.7–0.7) | 0.7  (0.7–0.7) | 99.6  (99.6–99.6) | 1.8  (1.1–2.8) | 2.0  (1.2–3.1) |
| ≥2 | 57.8  (56.9–58.8) | 58.9  (58.9–59.0) | 1.4  (1.4–1.4) | 0.7  (0.7–0.7) | 0.8  (0.8–0.8) | 99.6  (99.6–99.6) | 2.0  (1.3–3.0) | 2.1  (1.4–3.3) |
| ≥3 | 41.4  (40.5–42.4) | 75.6  (75.6–75.7) | 1.7  (1.7–1.7) | 0.8  (0.8–0.8) | 1.0  (0.9–1.0) | 99.6  (99.5–99.6) | 2.2  (1.4–3.4) | 2.4  (1.5–3.6) |
| ≥4 | 26.8  (26.0–27.7) | 86.4  (86.4–86.5) | 2.0  (1.9–2.0) | 0.9  (0.8–0.9) | 1.1  (1.1–1.2) | 99.5  (99.5–99.5) | 2.3  (1.5–3.7) | 2.5  (1.5–4.0) |
| ≥5 | 20.9  (20.2–21.7) | 90.5  (90.4–90.5) | 2.2  (2.1–2.3) | 0.9  (0.9–0.9) | 1.2  (1.2–1.3) | 99.5  (99.5–99.5) | 2.5  (1.5–4.1) | 2.6  (1.6–4.3) |

^a^ Adjusted for sex and age.

**STable 7. Associations between Columbia Suicide Severity Rating Scale Screen Version: Suicidal Ideation Severity Scale (past 1 month) and Suicide Behavior Scale (past 3 months) and suicide across diagnostic groups within ≤1 year follow-up. Associations expressed as odds ratios (OR) with 95% confidence intervals (CI).**

|  | **Suicidal Ideation Scale** | | | **Behavior Scale** | |
| --- | --- | --- | --- | --- | --- |
|  | Crude OR  (95% CI) | Adjusted OR (95% CI)^a^ | | Crude OR  (95% CI) | Adjusted OR (95% CI)^a^ |
| **Suicide ≤1 year** |  | |  |  |  |
| Anxiety disorders (F30-48) | 1.2 (0.9-1.4) | | 1.2 (1.0-1.5) | 2.1 (1.0-4.5) | 2.3 (1.1-5.1) |
| Mood disorders (F30-39) | 1.1 (0.9-1.3) | | 1.1 (0.9-1.3) | 1.6 (0.7-3.5) | 1.7 (0.8-3.7) |
| Substance use disorders (F10-19) | 1.1 (0.9-1.3) | | 1.1 (0.9-1.3) | 1.9 (1.0-3.9) | 2.2 (1.0-4.2) |
| ADHD (F90) | 1.2 (0.9-1.6) | | 1.2 (0.9-1.6) | 3.2 (1.1-9.2) | 3.7 (1.2-10.9) |
| Autism spectrum disorder (F84-89) | 1.2 (0.8-1.7) | | 1.2 (0.8-1.8) | 0.9 (0.2-6.5) | 0.9 (0.1-7.1) |
| Psychotic disorders (F20-29) | 1.3 (1.0-1.7) | | 1.3 (1.0-1.7) | 3.7 (1.4-9.4) | 3.7 (1.5-9.8) |
| Personality disorders (F60) | 1.2 (0.8-1.7) | | 1.2 (0.9-1.8) | 1.5 (0.4-5.8) | 1.5 (0.4-6.1) |

ADHD=Attention-deficit hyperactivity disorder.

^a^ Adjusted for sex and age.
